# Regulation of glycolysis in brown adipocytes by HIF-1α

**DOI:** 10.1038/s41598-017-04246-y

**Published:** 2017-06-22

**Authors:** Astrid L. Basse, Marie S. Isidor, Sally Winther, Nina B. Skjoldborg, Maria Murholm, Elise S. Andersen, Steen B. Pedersen, Christian Wolfrum, Bjørn Quistorff, Jacob B. Hansen

**Affiliations:** 1 Department of Biology, University of Copenhagen, Copenhagen, Denmark; 2 Department of Biomedical Sciences, University of Copenhagen, Copenhagen, Denmark; 3 Department of Clinical Medicine, Aarhus University, Aarhus, Denmark; 4 Department of Endocrinology and Internal Medicine, Aarhus University Hospital, Aarhus, Denmark; 5 Institute of Food, Nutrition and Health, ETH Zurich, Schwerzenbach, Switzerland

## Abstract

Brown adipose tissue takes up large amounts of glucose during cold exposure in mice and humans. Here we report an induction of glucose transporter 1 expression and increased expression of several glycolytic enzymes in brown adipose tissue from cold-exposed mice. Accordingly, these genes were also induced after β-adrenergic activation of cultured brown adipocytes, concomitant with accumulation of hypoxia inducible factor-1α (HIF-1α) protein levels. HIF-1α accumulation was dependent on uncoupling protein 1 and generation of mitochondrial reactive oxygen species. Expression of key glycolytic enzymes was reduced after knockdown of HIF-1α in mature brown adipocytes. Glucose consumption, lactate export and glycolytic capacity were reduced in brown adipocytes depleted of *Hif-1α*. Finally, we observed a decreased β-adrenergically induced oxygen consumption in *Hif-1α* knockdown adipocytes cultured in medium with glucose as the only exogenously added fuel. These data suggest that HIF-1α-dependent regulation of glycolysis is necessary for maximum glucose metabolism in brown adipocytes.

## Introduction

There are two main types of adipose tissue, white adipose tissue (WAT) and brown adipose tissue (BAT), which both store energy in the form of triglyceride. WAT mobilizes its triglyceride stores for the benefit of other tissues when energy supplies are scarce. BAT, on the other hand, utilizes stored triglyceride to fuel heat production. Upon cold exposure, sympathetic nerve fibers release noradrenaline at the surface of brown adipocytes, stimulating their β-adrenergic receptors. This stimulation leads to lipolysis and thermogenic gene expression. Long-chain fatty acids released by lipolysis activate the brown adipocyte-specific uncoupling protein 1 (UCP1). Activated UCP1 uncouples the proton gradient across the inner mitochondria membrane, thereby releasing its accumulated electrochemical energy as heat^1, 2^. The capacity of brown adipocytes to produce heat is termed thermogenic potential, and a β-adrenergically induced change in oxygen consumption is often used as a surrogate measure of this.

Following cold exposure of mice and humans, large amounts of glucose are taken up by BAT^3, 4^. Glucose uptake is mediated by glucose transport proteins (GLUTs), of which the two most prominent in adipose tissue are the insulin-independent GLUT1 and the insulin-dependent GLUT4^3, 5^. Glucose is metabolized to two molecules of pyruvate in glycolysis through 10 enzymatic steps^6^. Glycolysis can be divided into two parts: the first part is energy-consuming and converts glucose to two triose sugars; in the second part, energy is harvested by converting the triose sugars to pyruvate^6^. The first reaction of glycolysis is catalyzed by hexokinase (HK), which is the major regulator of glycolytic flux in cancer cells^7^. Of the four HK isoforms, at least HK2 is known to be expressed at high levels in adipose tissue^8^. A second key flux-controlling step is the phosphofructokinase (PFK) reaction. The final reaction of glycolysis is catalyzed by pyruvate kinase (PK). There are several isoforms of PK, including two muscle isoforms: muscle pyruvate kinase 1 and 2 (*Pkm1* and *Pkm2*). Studies with rodents have demonstrated a cold-induced increase in activity of HK, PFK and PK in BAT^9–11^. Consistent with this, we have reported increased expression of many glycolytic enzymes in BAT from cold-exposed mice^8^.

The transcription factor hypoxia inducible factor-1α (HIF-1α) induces the expression of most glycolytic enzymes during hypoxia. At normoxia, HIF-1α has a short half-life and its degradation is induced by prolyl-4-hydroxylases. During hypoxia, prolyl-4-hydroxylase activity is decreased and HIF-1α stability is increased^12^. At normoxia, HIF-1α activity has been suggested to depend not primarily on protein stability, but on transcriptional regulation of the *Hif-1α* gene^13^. HIF-1α forms a heterodimer with the more stable HIF-1β subunit, and the complex binds to hypoxic responsive elements to induce gene expression^12^. The role of HIF-1α in adipose tissue has been examined in several studies; however, whether HIF-1α has beneficial or detrimental functions is not clear. Some studies suggest that HIF-1α has effects in adipose tissue that negatively impact whole-body metabolism, e.g. by suppressing fatty acid oxidation and energy expenditure as well as by disturbing insulin sensitivity and glucose homeostasis^14–19^. Other mouse models suggest that adipose HIF-1α has beneficial effects on metabolic health, e.g. by protecting against high fat diet-induced obesity, insulin resistance and glucose intolerance as well as by augmenting mitochondrial biogenesis, energy expenditure and thermogenesis^20–22^. Thus, the functions of HIF-1α in adipose tissue are important and many.

Here we report the results of a comprehensive time course study of mice exposed to cold for up to eight days. Increased expression of *Glut1*, *Glut4* and several glycolytic enzymes was observed in interscapular BAT (iBAT). These changes were largely recapitulated in cultured brown adipocytes after β-adrenergic stimulation. In addition, expression of *Glut1* and several glycolytic enzymes was induced by hypoxia in brown adipocytes. Basal glycolytic gene expression as well as β-adrenergically- or hypoxia-induced increases in glycolytic gene expression was reduced by *Hif-1α* knockdown. In addition, knockdown of *Hif-1α* diminished glucose uptake, lactate secretion and glycolytic capacity as well as β-adrenergically stimulated oxygen consumption.

## Results

### Increased expression of genes encoding glucose transporters and glycolytic enzymes in BAT from cold-exposed mice

**Figure 1:**
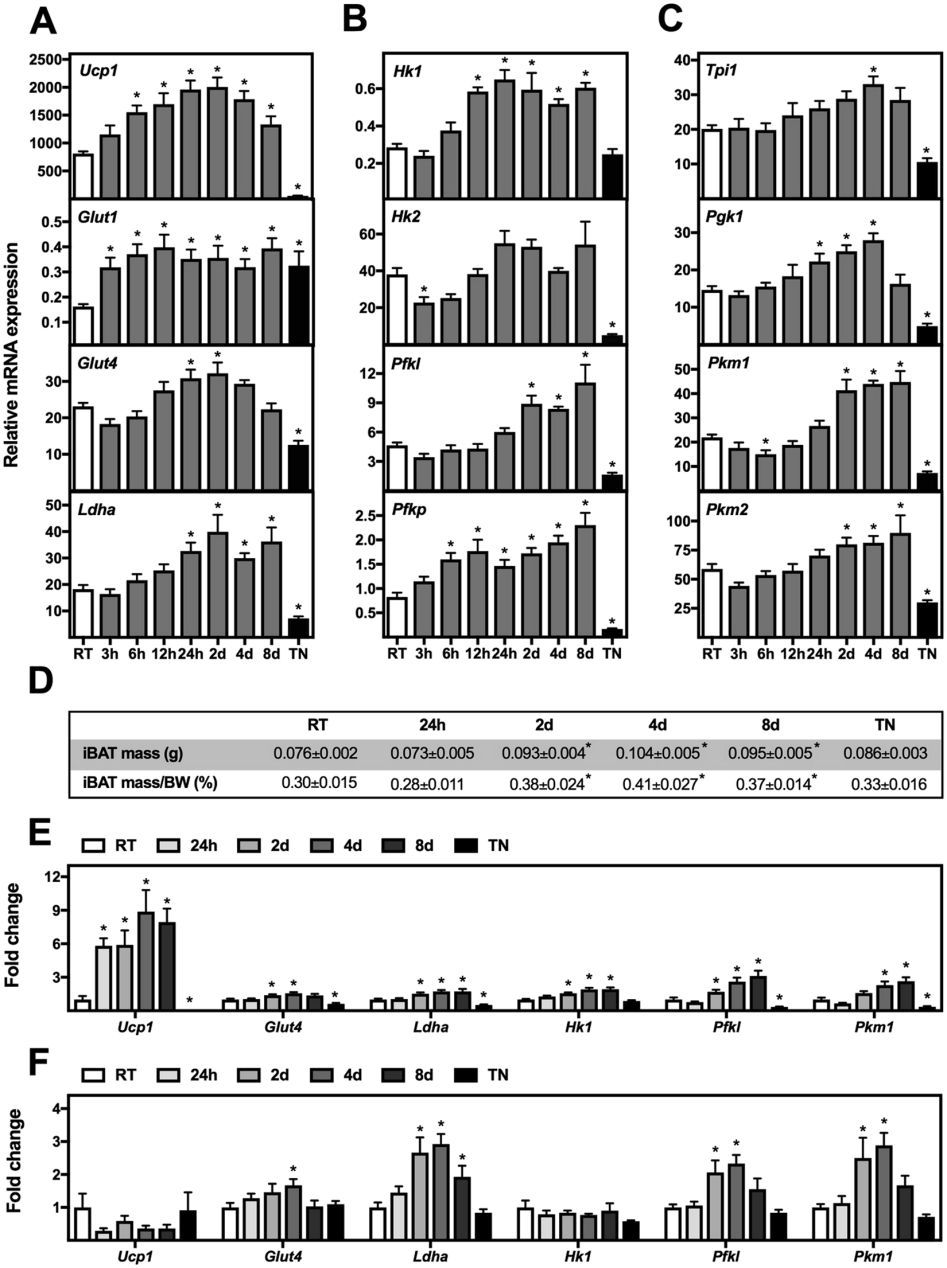
Expression levels of genes associated with glycolysis in iBAT, iWAT and eWAT of mice exposed to cold or thermoneutrality. Total RNA was isolated from iBAT, iWAT and eWAT of control mice kept at room temperature (RT, white bars) (n = 12), mice kept at thermoneutrality for 8 days (TN, black bars) (n = 6) and mice exposed to cold for 3 h, 6 h, 12 h, 24 h, 2 d, 4 d or 8 d (grey bars) (n = 6). Relative gene expression was measured in iBAT by RT-qPCR for: (**A**) *Ucp1*, *Glut1*, *Glut4* and *Ldha*; (**B**) *Hk1*, *Hk2*, *Pfkl* and *Pfkp*; (**C**) *Tpi1*, *Pgk1*, *Pkm1* and *Pkm2*. (**D**) Mass (g) of iBAT and iBAT mass as percent of body weight. (**E**) Fold change in gene expression level in iWAT and (**F**) eWAT. The mRNA expression levels were normalized to TATA-binding protein (*Tbp*). Data represents mean + SEM. *p < 0.05 *versus* RT.

To study the expression of glucose transporters and glycolytic enzymes in iBAT during cold exposure, mice kept at room temperature were transferred to 4 °C for 3 h, 6 h, 12 h, 24 h, 2 days, 4 days or 8 days. In addition, a group of mice were housed at thermoneutrality (30 °C) for 8 days. Gene expression was determined by RT-qPCR (Fig. 1A–C). *Ucp1* expression increased transiently, peaking after 2 days of cold exposure (Fig. 1A). Moreover, iBAT mass increased during cold exposure (Fig. 1D). Expression of the two glucose transporters *Glut1* and *Glut4* increased during the cold challenge (Fig. 1A). *Glut1* expression increased ~2-fold after 3 h in the cold and remained high throughout the study. We observed a bell-shaped expression profile for *Glut4* with a maximum induction of 1.4-fold after 2 days in the cold (Fig. 1A). We observed a ~2-fold decrease in *Glut4* expression of mice kept at thermoneutrality, a condition that unexpectedly increased *Glut1* expression ~2-fold (Fig. 1A).

Lactate production is an important anaerobic pathway linked to glycolysis^6^. Lactate dehydrogenase A (*Ldha*) is the *Ldh* isoform with the highest expression in iBAT (data not shown). *Ldha* expression was induced ~2-fold in mice exposed to cold for 1, 2, 4 or 8 days (Fig. 1A). Contrary, expression of *Ldha* was down-regulated ~2-fold in iBAT from mice kept at thermoneutrality.

Expression of several enzymes catalyzing the first (Fig. 1B) and second (Fig. 1C) part of glycolysis was increased progressively in iBAT during cold exposure. *Hk1* expression was increased ~2-fold from 12 h of cold exposure and onwards (Fig. 1B). Thermoneutrality did not affect *Hk1* expression. *Hk2* expression was significantly downregulated after 3 h of cold exposure and at thermoneutrality, but was otherwise not significantly affected by cold (Fig. 1B). *Pfkl* expression was induced ~2-fold in iBAT from mice exposed to cold for 2, 4 or 8 days (Fig. 1B). *Pfkp* expression increased more rapidly with a ~2-fold induction after 6 h and a maximum induction after 8 days cold exposure. Expression of *Pfkl* and *Pfkp* was decreased 2-3-fold in mice kept at thermoneutrality (Fig. 1B). Expression of triosephosphate isomerase 1 (*Tpi1*) and phosphoglycerate kinase 1 (*Pgk1*) increased modestly in a progressive manner during cold exposure with a significant maximum induction of 1.6- and 1.9-fold, respectively, at day 4 (Fig. 1C). Thermoneutrality caused a ~2-fold down-regulation of both. The expression profiles of *Pkm1* and *Pkm2* resembled that of *Pfkl*, except that *Pkm1* expression was significantly downregulated after 6 h in the cold (Fig. 1C). Overall these data show a general increase in expression of glycolytic enzymes in iBAT during cold exposure and a decrease in expression of the same genes at thermoneutrality compared to room temperature.

In inguinal and epididymal WAT, cold exposure induced the expression of *Glut4*, *Ldha*, *Pfkl* and *Pkm1* (Fig. 1E and F). *Ucp1* and *Hk1* mRNAs were selectively increased in inguinal compared with epididymal WAT.

### β-Adrenergic stimulation causes induction of several glycolytic enzymes in cultured brown adipocytes

**Figure 2:**
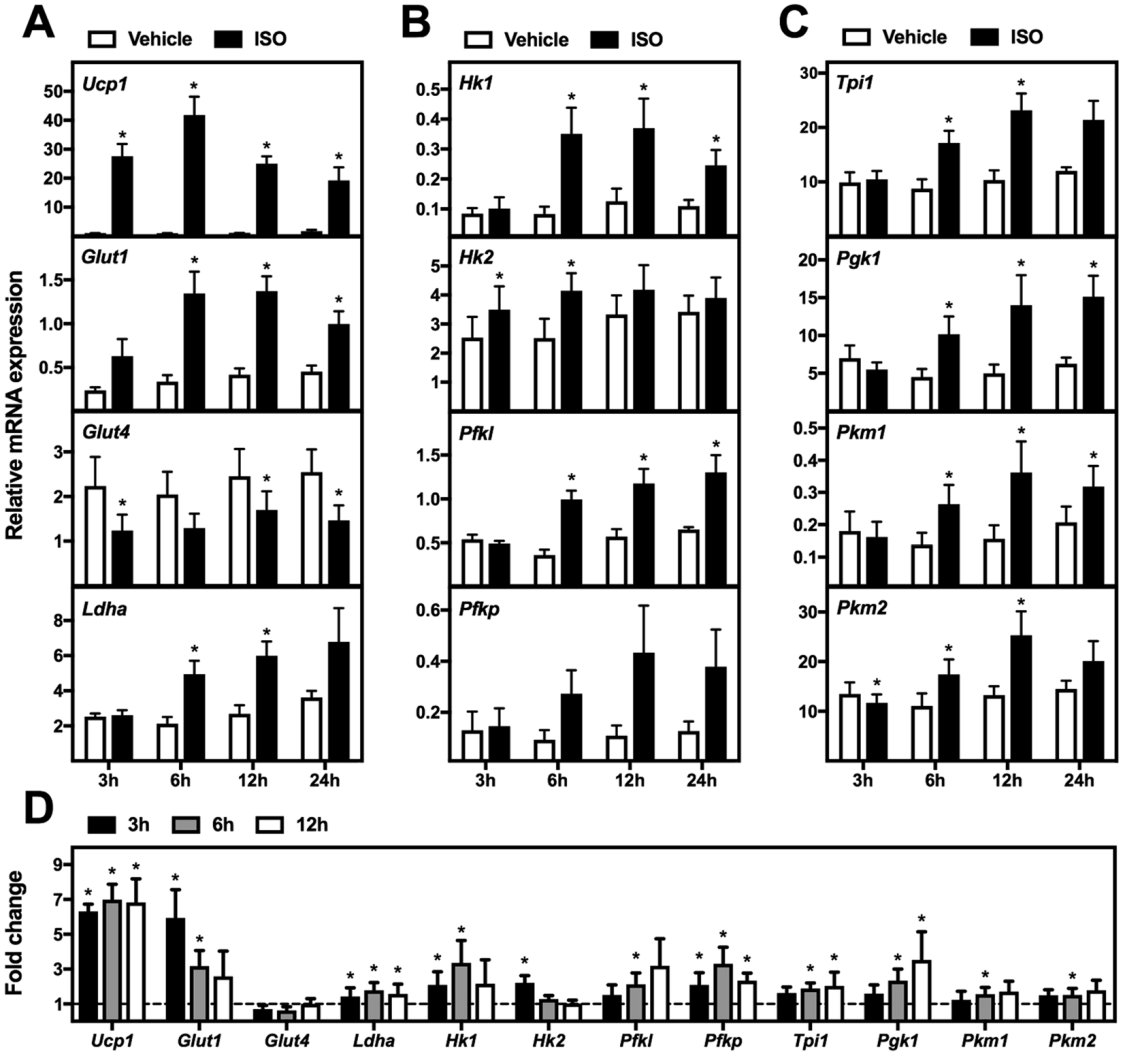
Expression levels of genes associated with glycolysis in brown adipocytes after β-adrenergic stimulation *in vitro*. (**A–C**) Mature brown adipocytes (WT-1) were treated with vehicle (white bars) or 0.1 μM isoproterenol (ISO, black bars) for 3, 6, 12 or 24 h. Total RNA was harvested and analyzed by RT-qPCR (n = 4). Relative gene expression was measured for: (**A**) *Ucp1*, *Glut1*, *Glut4* and *Ldha*; (**B**) *Hk1*, *Hk2*, *Pfkl* and *Pfkp*; (**C**) *Tpi1*, *Pgk1*, *Pkm1* and *Pkm2*. (**D**) Mature primary brown adipocytes were treated with vehicle or 0.1 μM isoproterenol for 3 h (black bars), 6 h (grey bars) or 12 h (white bars). Relative gene expression was measured and presented as fold change relative to the corresponding vehicle-treated samples (n = 4). mRNA expression levels were normalized to *Tbp*. Data represent mean of means + SEM. *p < 0.05 *versus* vehicle controls.

To test whether the expression of the glucose transporters and glycolytic enzymes was recapitulated in cultured brown adipocytes in response to β-adrenergic stimulation, we used the brown pre-adipocyte cell line WT-1^23^ and primary brown adipocytes. We stimulated mature WT-1 adipocytes at day 8 with the pan-β-adrenergic agonist isoproterenol (ISO) or vehicle for 3, 6, 12 or 24 h before harvesting. Expression of *Ucp1* was induced at all time points with a maximum induction of ~50-fold after 6 h (Fig. 2A). *Glut1* expression was significantly induced after ISO stimulation for 6, 12 and 24 h with maximal induction of 4-fold after 6 h (Fig. 2A). Contrary, ISO stimulation caused an up to 2-fold down-regulation of *Glut4* expression. Expression of *Ldha* was increased ~2-fold from 6 h of ISO stimulation (Fig. 2A).

The cold-induced changes in expression of glycolytic enzymes were to a large extent mimicked *in vitro* by β-adrenergic stimulation. The expression profile of *Hk1* was bell-shaped with a maximum ~4-fold induction after 6 h of ISO stimulation (Fig. 2B). *Hk2* expression was modestly increased after 3 and 6 h of stimulation. Expression of *Pfkl* and *Pfkp* was induced after 6, 12 and 24 h of stimulation with a maximum induction of ~3-fold, however, only the induction of *Pfkl* reached statistical significance (Fig. 2B). The enzymes catalyzing the second part of glycolysis followed a similar expression pattern with an induction after 6 h and a 2-3-fold increase in expression after 12 to 24 h of ISO stimulation (Fig. 2C).

To confirm these results in primary cells, we stimulated primary brown adipocytes with ISO for 3, 6 and 12 h (Fig. 2D). Expression of *Ucp1*, *Glut1* and *Ldha* was induced by ISO stimulation, together with the enzymes catalyzing the first part of the glycolysis. The enzymes catalyzing the second part of glycolysis followed a similar expression pattern with significant induction observed after 6 h of ISO stimulation (Fig. 2D).

Overall, this demonstrates a similar expression profile of glycolytic enzymes after cold exposure *in vivo* and β-adrenergic stimulation *in vitro*, although the kinetics of induction is faster *in vitro*.

### Mitochondrial uncoupling and hypoxia can induce the expression of glycolytic enzymes

**Figure 3:**
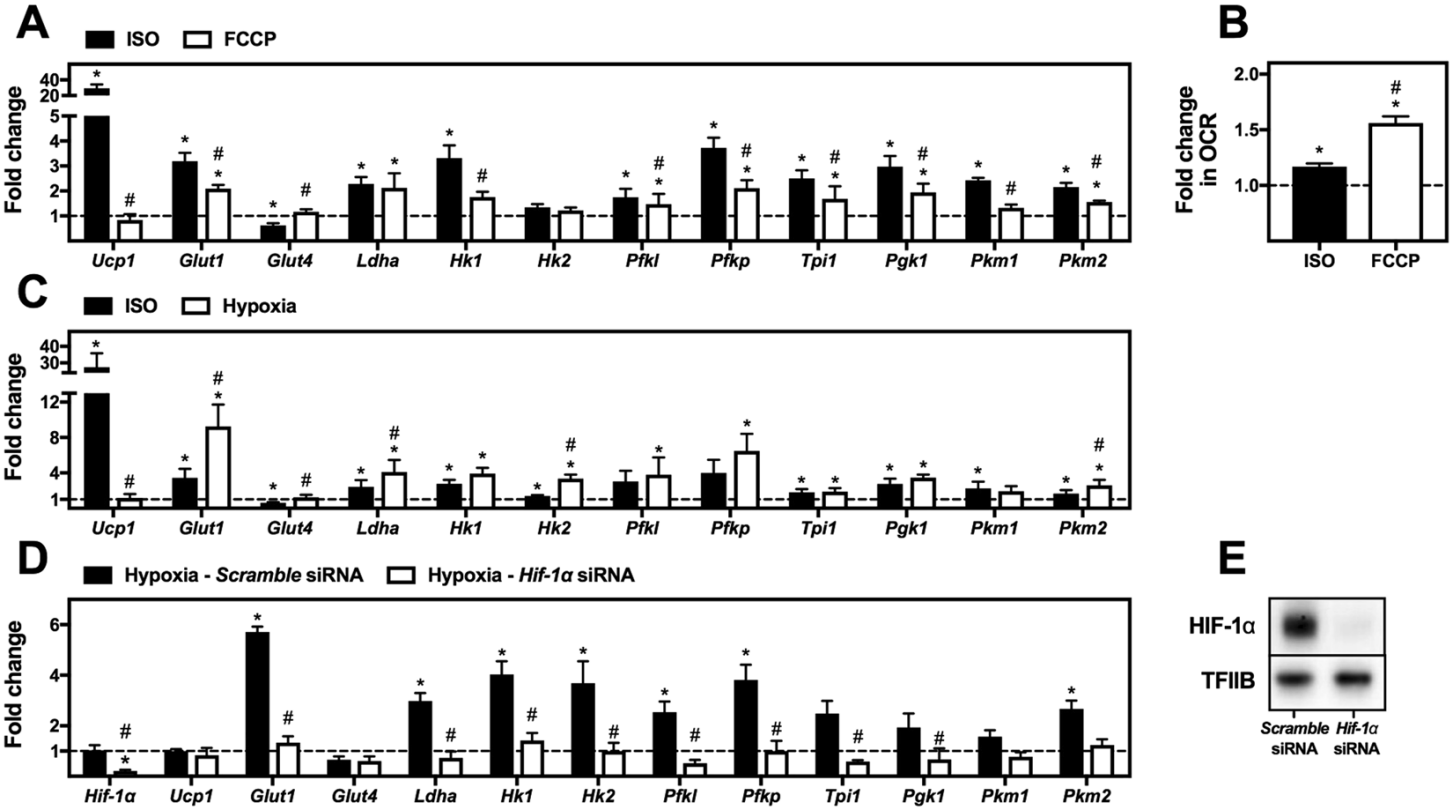
Expression levels of genes associated with glycolysis during chemical uncoupling or hypoxia. (**A**) Total RNA was isolated from mature brown adipocytes (WT-1) treated with vehicle, 0.1 μM isoproterenol (ISO) (black bars) or 1 μM of the chemical uncoupler FCCP (white bars) for 12 h. Relative gene expression was analyzed by RT-qPCR (n = 4) and presented as fold change relative to the corresponding vehicle-treated samples. (**B**) Representative fold induction in oxygen consumption rate (OCR) 30 min after stimulation of WT-1 brown adipocytes with 0.1 μM ISO (black bar) or 1 μM FCCP (white bar) on a Seahorse XF96 Flux Analyzer (n = 3) (**C**) Total RNA was isolated from mature brown adipocytes (WT-1) treated with vehicle or 0.1 μM ISO (black bars) or exposed to hypoxia (1% O_2_) (white bars) for 12 h. Relative gene expression was analyzed by RT-qPCR (n = 5) and presented as fold change relative to the corresponding control samples. (**D**) Total RNA was isolated from mature brown adipocytes (WT-1), which were reverse transfected with *scramble* siRNA or siRNA against *Hif-1α* at day 6 after initiation of differentiation. Mature cells at day 10 were exposed to normoxia or hypoxia (1% O_2_) for 12 h. Relative gene expression was analyzed by RT-qPCR (n = 3) and presented as fold change relative to the corresponding normoxia control samples. (**E**) Immunoblotting analyses of HIF-1α in brown WT-1 adipocytes transfected with *scramble* siRNA or siRNA against *Hif-1α*. The mRNA expression levels were normalized to *Tbp*. Gene expression data represent mean of means + SEM. *p < 0.05 *versus* vehicle control. ^#^p < 0.05 *versus* ISO stimulated cells (panel A and C) or *scramble* siRNA transfected cells (panel D). Transcription factor II B (TFIIB) was used as loading control in panel E. Full-length blots for panel E are shown in Supplementary Figure S1.

β-Adrenergic stimulation might induce the expression of glycolytic enzymes directly through signal transduction-dependent activation of transcription factors or indirectly through increased energy demands in response to the mitochondrial uncoupling. To distinguish between these two possibilities, WT-1 brown adipocytes were treated either with ISO or the chemical uncoupler FCCP (Fig. 3A). FCCP significantly increased expression of *Glut1* as well as *Ldha, Pfkl, Pfkp, Tpi1, Pgk1* and *Pkm2* compared to vehicle-treated cells (Fig. 3A). However, FCCP was a less potent inducer of glycolytic gene expression than ISO, even though experiments on a Seahorse XF96 Flux Analyzer established that 1 µM FCCP was sufficient to cause a more pronounced mitochondrial uncoupling than 0.1 µM ISO in these cells (Fig. 3B). Thus, these data indicate that the increased energy demand after mitochondrial uncoupling can explain some, but not all of the increased expression of glycolytic enzymes in response to β-adrenergic stimulation.

One mechanism by which mitochondrial uncoupling might induce changes in expression of the glycolytic genes is through hypoxia. To test whether hypoxia mimicked the effect on glycolytic gene expression caused by ISO stimulation, we exposed cells to hypoxia (1% O_2_) or ISO for 12 h. Hypoxia caused a significant induction of *Glut1*, *Ldha*, *Hk1*, *Hk2, Pfkl, Pfkp, Tpi1*, *Pgk1* and *Pkm2* (Fig. 3C). The expression of *Hk1*, *Pfkl*, *Pfkp, Tpi1*, *Pgk1*, *Pkm1* and *Pkm2* was induced to a similar level by hypoxia and ISO stimulation (Fig. 3C). This indicates that hypoxia in response to uncoupling might induce glycolytic gene expression in response to ISO stimulation.

The most likely candidate regulating glycolytic gene expression in response to hypoxia is HIF-1α. To test whether HIF-1α is responsible for regulating glycolytic gene expression in brown adipocytes in response to hypoxia, we knocked down *Hif-1α* by siRNA in mature WT-1 adipocytes. Four days after siRNA transfection, the cells were exposed to normoxia or hypoxia for 12 h, and gene expression was analyzed. The siRNA reduced both *Hif-1α* mRNA (~80%) (Fig. 3D) and HIF-1α protein levels (Fig. 3E). The induction of glycolytic gene expression by hypoxia was completely blunted by knockdown of *Hif-1α* (Fig. 3D). This shows that HIF-1α is responsible for hypoxia-induced expression of the glycolytic enzymes in brown adipocytes.

### Regulation of HIF-1α levels in cultured brown adipocytes

**Figure 4:**
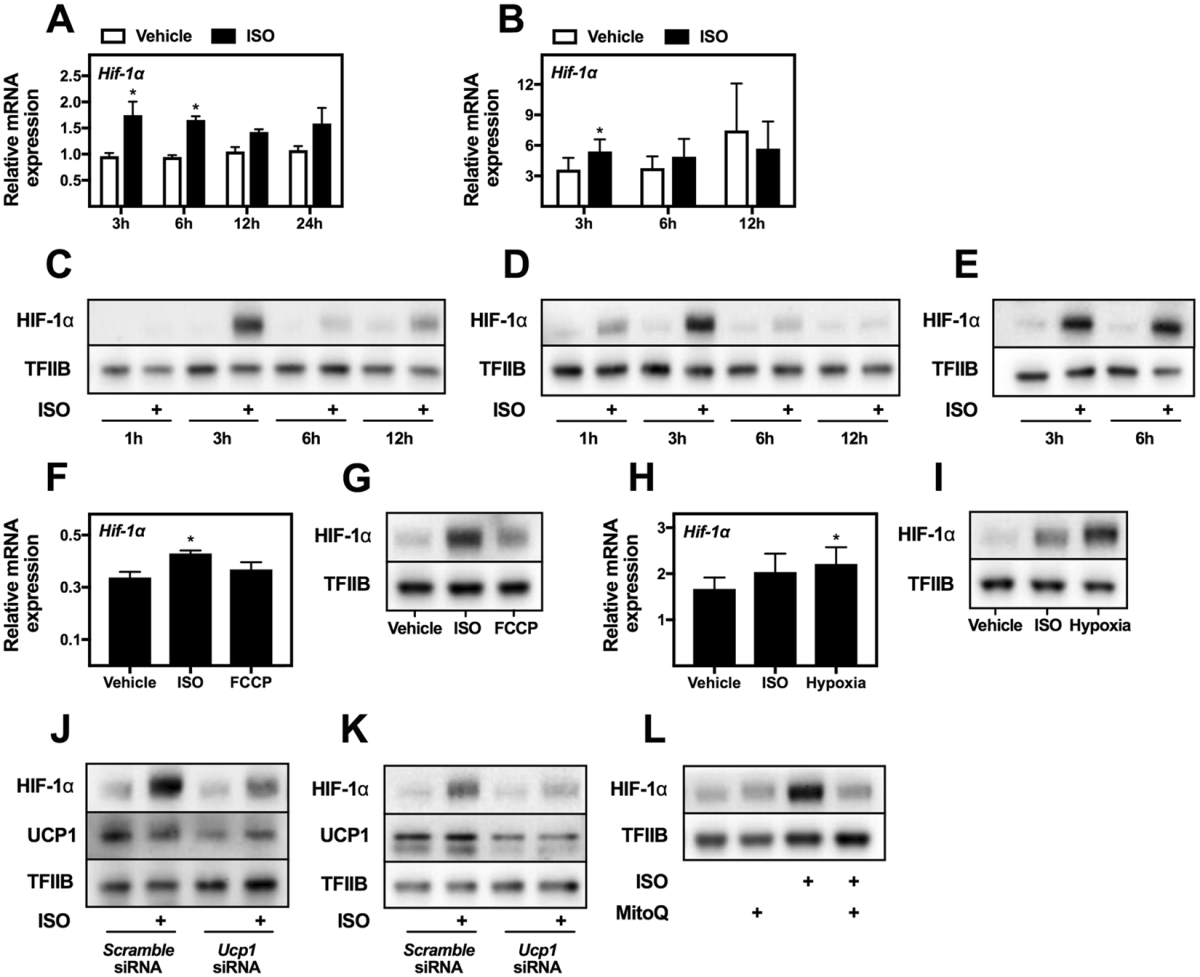
*Hif-1α* mRNA and HIF-1α protein levels. (**A**) Total RNA was isolated from mature brown adipocytes (WT-1). Relative mRNA expression was measured by RT-qPCR for *Hif-1α* in cells treated with vehicle (white bars) or 0.1 μM ISO (black bars) for 3, 6, 12 or 24 h (n = 4). (**B**) Total RNA was isolated from primary mature brown adipocytes. Relative mRNA expression was measured by RT-qPCR for *Hif-1α* in cells treated with vehicle (white bars) or 0.1 μM ISO (black bars) for 3, 6 or 12 h (n = 4). Immunoblotting analysis of HIF-1α in (**C**) WT-1 brown adipocytes and in (**D**) primary brown adipocytes stimulated with vehicle or ISO for 1, 3, 6 and 12 h. (**E**) Immunoblotting analysis of HIF-1α in human brown adipocytes stimulated with vehicle or ISO for 3 or 6 h. (**F**) Relative gene expression for *Hif-1α* (n = 4) and (**G**) immunoblotting analyses of HIF-1α were performed on mature brown adipocytes (WT-1) treated with vehicle, 0.1 μM ISO or 1 μM FCCP for 12 h (gene expression) or 4 h (immunoblotting). (**H**) Relative gene expression for *Hif-1α* (n = 4) and (**I**) immunoblotting analysis of HIF-1α were performed on mature brown adipocytes (WT-1) treated with vehicle, 0.1 μM ISO or exposed to hypoxia (1% O_2_) for 12 h (gene expression) or 3 h (immunoblotting). (**J**) Immunoblotting analysis for HIF-1α and UCP1 was performed on mature brown adipocytes (WT-1) or (**K**) in mature primary brown adipocytes, which were reverse transfected with a *scramble* siRNA or siRNAs against *Hif-1α* at day 6 after initiation of differentiation or 8 days after isolation, respectively. Four days later, the adipocytes were stimulated with vehicle or 0.1 μM ISO for 3 h. mRNA levels were normalized to *Tbp*. (**L**) Immunoblotting analysis for HIF-1α was performed on mature brown adipocytes (WT-1), which were pretreated or not with 5 μM MitoQ for 1 h, followed by stimulation with vehicle or 0.1 μM ISO for an additional 3 h. Data represent mean of means + SEM. *p < 0.05 *versus* vehicle (panel A, B, F and H). TFIIB was used as loading control for immunoblotting (panel C–E, G and I–L). Full-length blots for panels C–E, G and I–L are shown in Supplementary Figure S1.

The HIF-1α-dependent induction of many glycolytic genes after hypoxia prompted us to measure levels of HIF-1α in response to β-adrenergic stimulation of cultured brown adipocytes. Increased *Hif-1α* mRNA levels were detected in WT-1 (Fig. 4A) and primary brown adipocytes (Fig. 4B) in response to ISO stimulation. Immunoblotting demonstrated a transient accumulation of HIF-1α protein in response to ISO stimulation in WT-1 (Fig. 4C) and primary brown adipocytes (Fig. 4D). The changes in HIF-1α protein levels were much more pronounced than *Hif-1α* mRNA levels, indicating increased stability of HIF-1α in response to ISO stimulation. HIF-1α protein levels also accumulated in a human brown adipocyte cell model after ISO stimulation (Fig. 4E).

Treatment with FCCP did not increase *Hif-1α* mRNA levels in WT-1 adipocytes, but caused a modest increase in HIF-1α protein levels (Fig. 4F and G). Hypoxia increased both *Hif-1α* mRNA and HIF-1α protein levels in WT-1 adipocytes (Fig. 4H and I). To study the importance of uncoupling for HIF-1α stability further, we knocked down *Ucp1* expression in mature WT-1 adipocytes and in mature primary adipocytes with siRNA (Fig. 4J and K). Four days after siRNA transfection, the cells were stimulated with vehicle or ISO for 3 h, and protein levels were analyzed. Knockdown of *Ucp1* blunted the increase in HIF-1α levels after ISO stimulation in both WT-1 and primary adipocytes (Fig. 4J and K). Of notice, even without exposure to ISO, FCCP or hypoxia, low levels of HIF-1α protein were present in human and mouse brown adipocytes (Fig. 4C–E,G,I and J–L). Altogether, these results highlight the importance of UCP1, and thereby mitochondrial uncoupling, for ISO-induced HIF-1α stabilization. However, the modest effect of FCCP compared to ISO on HIF-1α stabilization (Fig. 4G) indicates that uncoupling-induced hypoxia is not the only mechanism through which β-adrenergic stimulation stabilizes HIF-1α. It was recently demonstrated that β-adrenergic stimulation induced production of mitochondrial reactive oxygen species (ROS) in brown adipocytes^24^. Since ROS is capable of stabilizing HIF-1α in a human hepatoblastoma cell line^25^, we hypothesized that β-adrenergic stimulation could also stabilize HIF-1α through increased ROS production. To test this, we pretreated WT-1 adipocytes with the mitochondria-targeted antioxidant MitoQ for 1 h, followed by stimulation with ISO or vehicle for an additional 3 h (Fig. 4L). MitoQ treatment abolished the β-adrenergically induced stabilization of HIF-1α. This indicates that mitochondrial ROS production plays an essential role in stabilizing HIF-1α in response to β-adrenergic stimulation.

### Regulation of basal and β-adrenergically induced glycolytic gene expression by HIF-1α in cultured brown adipocytes

**Figure 5:**
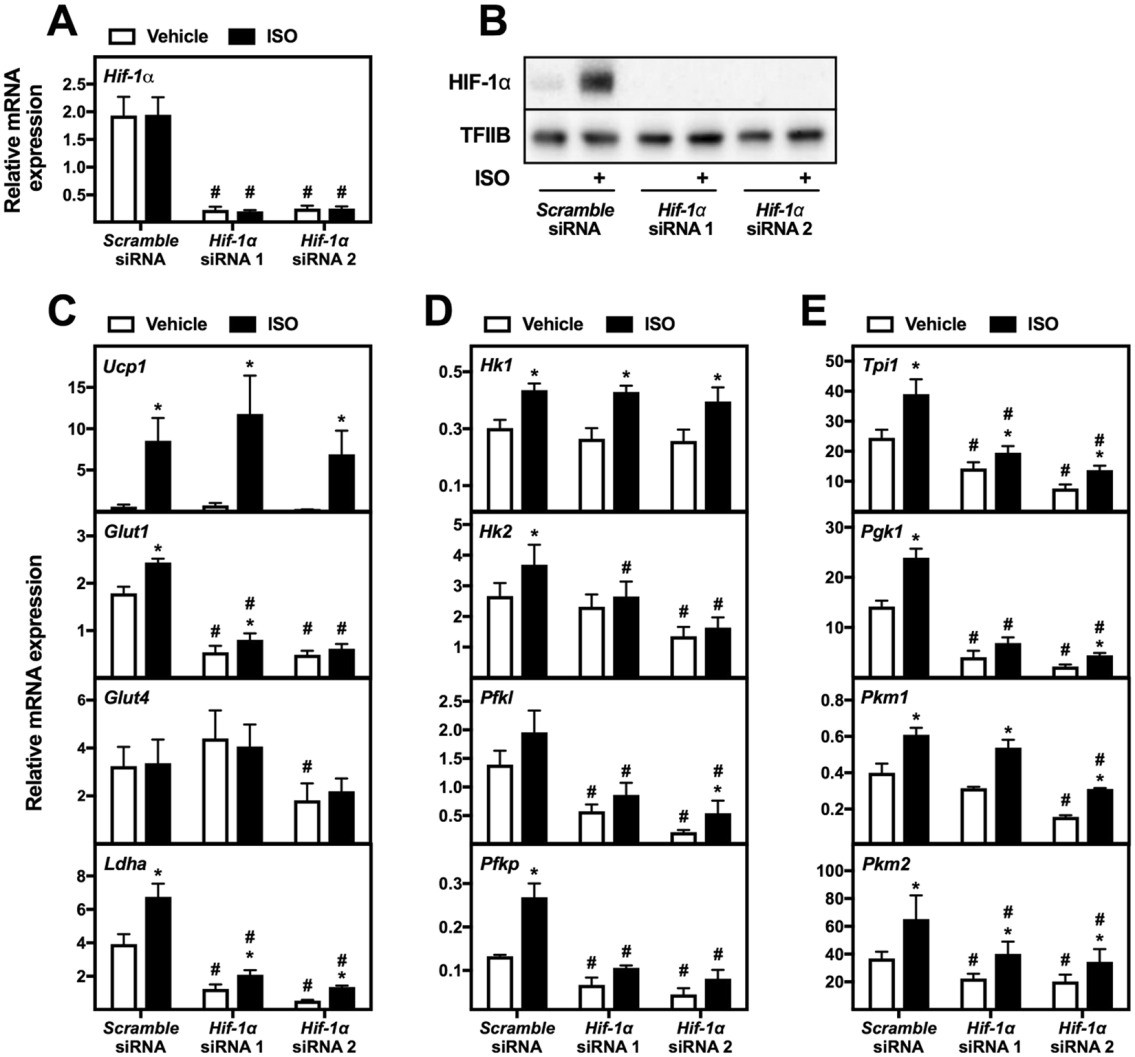
Involvement of HIF-1α in regulation of expression of genes associated with glycolysis in a brown adipocyte cell line. Mature brown adipocytes (WT-1) were reverse transfected with two different siRNAs against *Hif-1α* at day 6 after initiation of differentiation. Mature cells at day 10 were treated with vehicle (white bars) or 0.1 μM ISO (black bars) for 12 h. Total RNA was harvested and analyzed by RT-qPCR (n = 3). Relative gene expression was measured for: (**A**) *Hif-1α*; (**C**) *Ucp1*, *Glut1*, *Glut4* and *Ldha*; (**D**) *Hk1*, *Hk2*, *Pfkl* and *Pfkp*; (**E**) *Tpi1*, *Pgk1*, *Pkm1* and *Pkm2*. mRNA expression levels were normalized to *Tbp*. Data represent mean of means + SEM. *p < 0.05 *versus* vehicle-treated cells transfected with the same siRNA. ^#^p < 0.05 *versus scramble* siRNA transfected cells treated in the same way (vehicle or ISO). (**B**) Immunoblotting analysis of HIF-1α in brown adipocytes (WT-1) reverse transfected with *scramble* siRNA or two different siRNAs against *Hif-1α*. Four days after the transfection, adipocytes were treated with vehicle or 0.1 μM ISO for 3 h. TFIIB was used as loading control. Full-length blots for panel B are shown in Supplementary Figure S1.

To investigate the involvement of HIF-1α in basal and β-adrenergically induced glycolytic gene expression, we knocked down *Hif-1α* in mature WT-1 adipocytes with two different siRNAs (siRNA 1 and 2). Four days post-transfection, the cells were stimulated with vehicle or ISO for 12 h, and gene expression was analyzed. The two siRNAs reduced both *Hif-1α* mRNA (~90%) (Fig. 5A) and HIF-1α protein levels (Fig. 5B). Knockdown of *Hif-1α* with siRNA 1 and/or 2 reduced basal expression of *Glut1, Glut4, Ldha* (Fig. 5C) and all the glycolytic enzymes measured, except *Hk1* (Fig. 5D and E). Similarly, *Hif-1α* knockdown with siRNA 1 and/or 2 diminished expression of the same set of genes, excluding *Glut4*, after ISO stimulation (Fig. 5C–E). Finally, ISO stimulation did no longer significantly induce expression of *Hk2* and *Pfkp* after *Hif-1α* knockdown with siRNA 1 and/or 2 (Fig. 5C). Of notice, basal expression of the glycolytic genes was consistently higher when cells were transfected with siRNA compared to the non-transfected cells in Fig. 2. This might be due to several factors: the cells being replated during differentiation; the cells being cultured in 96-well format compared to the larger well formats used in all non-siRNA experiments; or the transfection procedure itself. Due to this higher basal expression, the ISO-induced expression of glycolytic genes was less pronounced in the experiments where the cells were siRNA-transfected.

**Figure 6:**
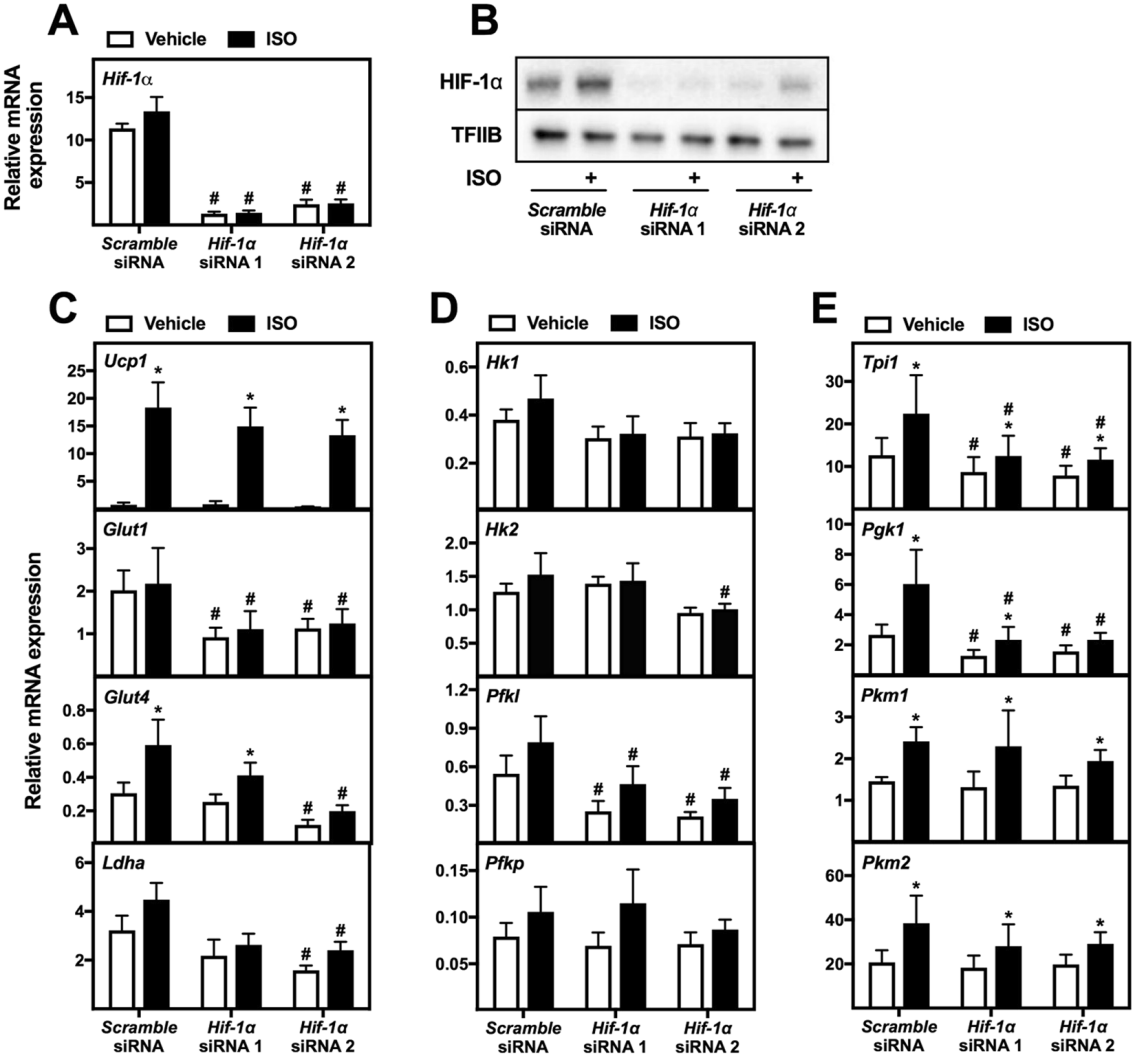
Involvement of HIF-1α in regulation of expression of genes associated with glycolysis in primary brown adipocytes. Primary mature brown adipocytes were reverse transfected with *scramble* siRNA or two different siRNAs against *Hif-1α* at day 8 after isolation. Four days later the primary adipocytes were treated with vehicle (white bars) or 0.1 μM ISO (black bars) for 12 h. Total RNA was harvested and analyzed by RT-qPCR (n = 4). Relative gene expression was measured for: (**A**) *Hif-1α*; (**C**) *Ucp1*, *Glut1*, *Glut4*, and *Ldha*; (**D**) *Hk1*, *Hk2*, *Pfkl* and *Pfkp*; (**E**) *Tpi1*, *Pgk1*, *Pkm1* and *Pkm2*. The mRNA expression levels were normalized to *Tbp*. Data represent mean of means + SEM. *p < 0.05 *versus* vehicle-treated cells transfected with the same siRNA. ^#^p < 0.05 *versus scramble* siRNA transfected cells treated in the same way (vehicle or ISO). (**B**) Immunoblotting analysis of HIF-1α in primary brown adipocytes transfected with *scramble* siRNA or two different siRNAs against *Hif-1α*. Four days after transfection, adipocytes were treated with vehicle or 0.1 μM ISO for 3 h. TFIIB was used as loading control. Full-length blots for panel B are shown in Supplementary Figure S1.

To validate the results from WT-1 cells, *Hif-1α* was also knocked down in primary brown adipocytes. Mature primary brown adipocytes were transfected 8 days after isolation with *scramble* siRNA or the two siRNAs against *Hif-1α*. Four days later, the cells were stimulated with vehicle or ISO for 12 h, and gene expression was analyzed. *Hif-1α* expression was reduced with ~90% and ~80% by siRNA 1 and 2, respectively (Fig. 6A), and the protein levels of HIF-1α was reduced as well (Fig. 6B). Knockdown of *Hif-1α* with siRNA 1 and/or 2 reduced the basal expression of *Glut1*, *Glut4*, *Ldha*, *Pfkl*, *Tpi1* and *Pgk1* (Fig. 6C–E). After ISO stimulation, the expression of *Glut1, Glut4, Ldha, Hk2, Pfkl, Tpi1* and *Pgk1*was reduced by *Hif-1α* knockdown compared to control cells (Fig. 6C–E).

In summary, HIF-1α is important for basal expression of glycolytic genes as well as their expression after β-adrenergic stimulation in both primary and immortalized brown adipocytes.

### HIF-1α promotes glycolysis and β-adrenergically stimulated thermogenesis in cultured brown adipocytes

**Figure 7:**
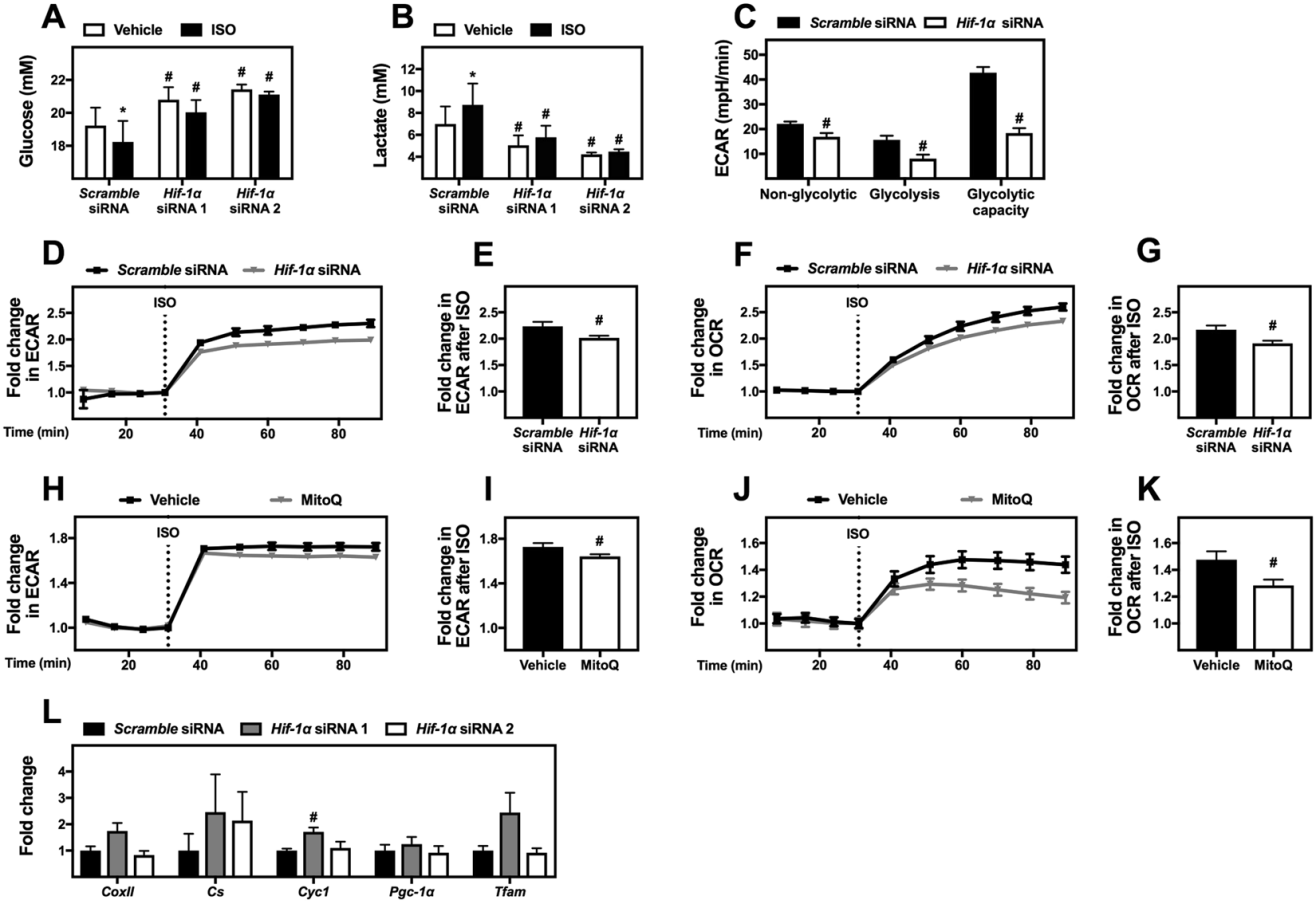
The importance of HIF-1α for β-adrenergically stimulated glycolysis and thermogenesis. Mature brown adipocytes (WT-1) were reverse transfected with *scramble* siRNA or two different siRNAs against *Hif-1α*. Four days later, the adipocytes were stimulated with vehicle or 1 μM ISO for 6 h, after which glucose (**A**) and lactate (**B**) measurements were performed on the medium. (**C**) Representative results from Seahorse XF Glycolysis Stress Tests performed on mature adipocytes (WT-1), which were reverse transfected with *scramble* siRNA or an siRNA against *Hif-1α* (siRNA 1) (n = 3). (**D–G**) Extracellular acidification rate (ECAR) and oxygen consumption rate (OCR) were measured four days after transfection (n = 5). (**D**) Representative time-course of ECAR during basal conditions and after injection of 1 μM ISO. (**E**) Fold change in ECAR between the basal level and the measurement 30 min after ISO stimulation. (**F**) Representative time-course of OCR during basal conditions and after injection of 1 μM ISO. (**G**) Fold change in OCR between the basal level and the measurement 30 min after ISO stimulation. (**H–K**) ECAR and OCR were measured at day 8 on a Seahorse XF96 Flux Analyzer after treatment with 5 μM MitoQ or vehicle (n = 4). (**H**) Representative time-course of ECAR during basal conditions and after injection of 1 μM ISO. (**I**) Fold change in ECAR between the basal level and the measurement 30 min after ISO stimulation. (**J**) Representative time-course of OCR during basal conditions and after injection of 1 μM ISO. (**K**) Fold change in OCR between the basal level and the measurement 30 min after ISO stimulation. (**L**) Total RNA was isolated from mature adipocytes (WT-1) transfected with *scramble* siRNA or two different siRNAs against *Hif-1α*. Relative gene expression was analyzed by RT-qPCR and presented as fold change relative to the *scramble* siRNA-transfected cells. The genes measured were *Cyc1*, *CoxII*, *Cs, Pgc-1α* and *Tfam* (n = 3). mRNA expression levels were normalized to *Tbp*. Data represent mean + SEM (panel C-K) or mean of means + SEM (panel A, B and L). *p < 0.05 *versus* vehicle-treated cells. ^#^p < 0.05 *versus scramble* siRNA transfected cells.

Next, we examined whether HIF-1α influenced glucose consumption, lactate secretion and glycolysis in cultured brown adipocytes. Glucose consumption and lactate secretion were determined by measuring their concentrations in the cell culture medium. To determine the impact of HIF-1α, *Hif-1α* was knocked down in WT-1 brown adipocytes at day 6 (on average 83% knockdown at the mRNA level, data not shown). Four days after siRNA transfection, glucose uptake and lactate secretion was determined after 6 h of treatment with vehicle or ISO (Fig. 7). *Hif-1α* knockdown significantly decreased basal glucose uptake (Fig. 7A). Silencing of *Hif-1α* expression also diminished glucose uptake during 6 h of ISO stimulation (Fig. 7A). Furthermore, *Hif-1α* knockdown significantly decreased lactate secretion during exposure to both vehicle (basal secretion) and β-adrenergic stimulation, suggesting an attenuated glycolytic flux (Fig. 7B). To elaborate on the effect of HIF-1α on glycolysis under basal conditions we performed a glycolysis stress test on mature adipocytes transfected with either *scramble* siRNA or *Hif-1α* siRNA (Fig. 7C). Knockdown of *Hif-1α* decreased non-glycolytic ECAR, glycolysis and glycolytic capacity. These results highlight the importance of HIF-1α for glycolysis in brown adipocytes under basal conditions.

This prompted us to measure the effect of *Hif-1α* knockdown on ISO-stimulated glycolysis and thermogenesis using cell culture medium containing 5 mM glucose as the only exogenously added fuel. ECAR was measured under basal conditions and after addition of 1 μM ISO (Fig. 7D and E). Knockdown of *Hif-1α* (on average 93% knockdown at the mRNA level, data not shown) reduced ISO-stimulated ECAR by 22% (Fig. 7D and E). β-Adrenergically stimulated OCR was reduced by 18% in response to *Hif-1α* knockdown in the same experiment (Fig. 7F and G). To further investigate the importance of mitochondrial ROS production for the HIF-1α-dependent effects on glucose metabolism in response to β-adrenergic stimulation, we measured the effect of MitoQ treatment on ISO-stimulated glycolysis and thermogenesis using the same conditions as above (Fig. 7H–K). MitoQ treatment reduced β-adrenergically stimulated ECAR by 12% (Fig. 7H and I) and OCR by 40% (Fig. 7J and K). This indicates that mitochondrial ROS production is even more important for β-adrenergically stimulated thermogenesis than *Hif-1α* knockdown. Contrary, mitochondrial ROS production was less important for ISO-induced glucose metabolism than HIF-1α depletion.

The decrease in OCR after knockdown of *Hif-1α* might not necessarily be due to changes in glycolytic flux, but could be due to dysregulated expression of mitochondrial genes, since mice expressing an adipose-specific dominant negative form of HIF-1α display decreased mitochondrial gene expression^21^. However, we did not observe significantly decreased expression of the mitochondrial genes cytochrome *c* oxidase subunit 2 (*CoxII*), citrate synthase (*Cs*), cytochrome c-1 (*Cyc1*), peroxisome proliferator-activated receptor gamma coactivator-1α (*Pgc-1α*) and mitochondrial transcription factor A (*Tfam*) four days after *Hif-1α* knockdown in mature WT-1 adipocytes (Fig. 7L). Overall, these results emphasize an important role of HIF-1α in controlling the expression of glycolytic enzymes and glycolysis in brown adipocytes *in vitro*.

## Discussion

An inverse correlation between BAT activity and blood glucose concentrations in humans has been reported^26^, suggesting a potentially important role for BAT in glucose homeostasis, and BAT transplants have been shown to diminish high fat diet-induced insulin resistance in mice^27^. We have previously shown an increased expression of enzymes linked to glucose metabolism in BAT in response to cold^8^. In the present study, we have investigated the transcriptional regulation of glycolytic gene expression and its importance for glucose consumption and glycolysis in brown adipocytes.

Cold exposure and β-adrenergic stimulation cause an increase in glucose uptake in BAT^3, 4^. Glucose uptake in brown adipocytes is believed to be mediated primarily by GLUT1 and GLUT4. GLUT1 translocate to the plasma membrane after β-adrenergic stimulation of cultured brown adipocytes^5, 28, 29^, and both cold-induced up- and down-regulation of *Glut1* in BAT have been reported^3, 30^. GLUT4 levels and translocation to the plasma membrane increase after cold exposure *in vivo* ^31^. Here, we observed increased expression of both *Glut1* and *Glut4* in iBAT of mice exposed to cold (Fig. 1). The increase in *Glut1* expression was recapitulated in β-adrenergically stimulated cultured brown adipocytes, however, this was not the case for *Glut4* (Fig. 2). The inverse regulation of *Glut1* and *Glut4* expression after β-adrenergic stimulation of cultured brown adipocytes has been reported by others^5^.

We observed increased expression of the enzymes catalyzing both the first and second part of glycolysis in iBAT of mice exposed to cold and in cultured brown adipocytes stimulated with a β-adrenergic agonist (Figs 1 and 2). The increased expression of enzymes catalyzing the second part of glycolysis indicates an increased flow all the way through glycolysis and not only through the first 4 steps, which are required for conversion of glucose into glycerol. A high glycolytic flux in BAT is supported by the prominent expression of the *Pkm2* isoform (Fig. 1) since its activity is induced allosterically by positive feedback loops when glycolytic flux is high^32^. A substantial flow through glycolysis after cold exposure is also supported by studies demonstrating increased glucose conversion to CO_2_ in BAT of cold-exposed mice and in brown adipocytes after β-adrenergic stimulation^33–35^.

The increased expression of *Ldha* after cold exposure or β-adrenergic stimulation (Figs 1 and 2) and the increased lactate release in response to β-adrenergic stimulation imply increased reduction of pyruvate to lactate. A high degree of lactate production in BAT is emphasized by a study in rats in which lactate and pyruvate release accounted for 33% to 88% of the glucose taken up by iBAT after stimulation with increasing doses of noradrenaline^36^. Moreover, overexpression of UCP1 or treatment with FCCP induces an increased glycolytic flux and an increased lactate production in white adipocytes^33^. Accordingly, we observed increased expression of *Ldha* in response to FCCP treatment. This indicates that mitochondrial uncoupling by itself induces lactate production.

Chronic treatment with FCCP has been reported to induce glucose uptake and glycolytic flux in white adipocytes^33^. Accordingly, β-adrenergically induced glucose uptake in BAT appears to depend on uncoupling since β-adrenergic stimulation does not induce glucose uptake in BAT of *Ucp1* knockout mice^29^, although this observation is still a matter of discussion^34^. However, since treatment with FCCP only partly phenocopied the effect of ISO stimulation (Figs 3 and 4), this points to the existence of an energy sensing-independent pathway regulating the expression of glycolytic enzymes after β-adrenergic stimulation.

**Figure 8:**
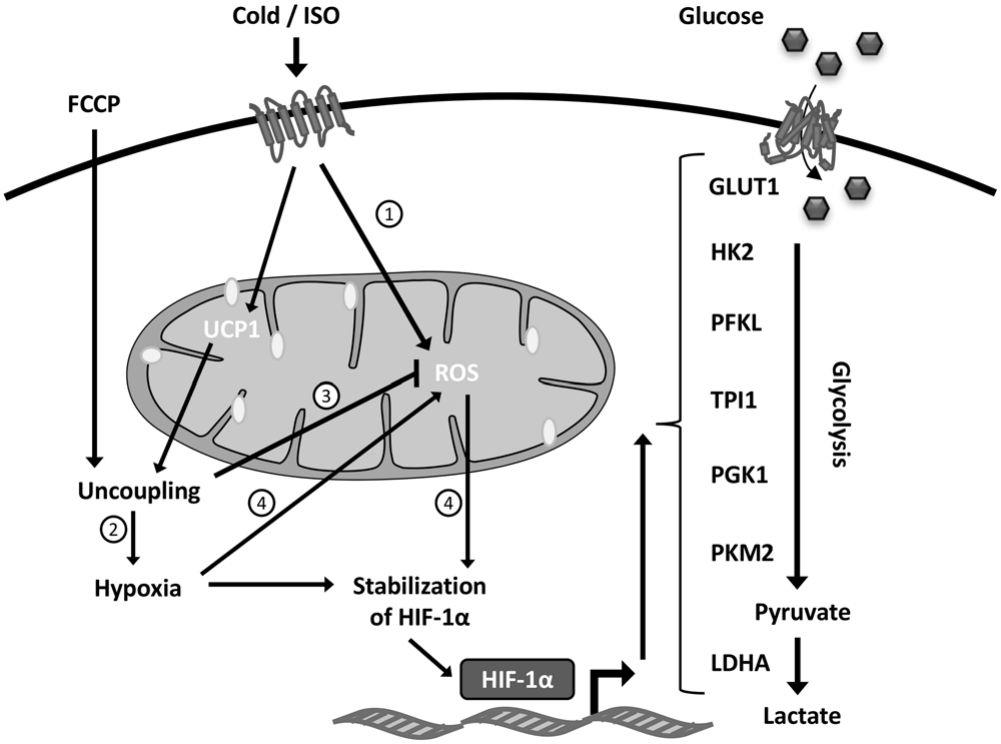
A proposed model for HIF-1α-dependent regulation of glycolysis in brown adipocytes. Illustrated is a model of how cold and β-adrenergic stimulation cause HIF-1α stabilization in brown adipocytes through mitochondrial uncoupling and reactive oxygen species (ROS) production. HIF-1α in turn induces the expression of *Glut1, Hk2, Pfkl, Tpi1, Pgk1, Pkm2* and *Ldha*, increasing glycolytic flux capacity. **1**) β-Adrenergic stimulation induces the production of mitochondrial ROS in brown adipocytes^24^. **2**) Cold-stimulated mitochondrial uncoupling induces hypoxia in BAT^41^. **3**) FCCP-induced mitochondrial uncoupling decreases mitochondrial ROS production in brown adipocytes^42, 43^. **4**) Hypoxia-induced HIF-1α stabilization through increased mitochondrial ROS production^25^.

Transcriptional regulation of glycolytic genes has primarily been studied in relation to cancer. One of the best-described transcription factors controlling glycolytic gene expression in cancer cells is HIF-1α^37^. HIF-1α activity is mainly regulated post-transcriptionally in response to hypoxia. To this end, we observed increased HIF-1α protein levels after ISO stimulation in primary and immortalized brown adipocytes (Fig. 4). The increase in HIF-1α levels was more pronounced at protein level compared to mRNA level. Although clearly elevated after ISO stimulation, the HIF-1α protein was also detectable in untreated brown adipocytes (Figs 4–6). Hypoxia is induced by cold in BAT of lean mice, as observed by pimonidazole staining, and the cold-induced hypoxia is dependent on mitochondrial uncoupling, since it is blunted in *Ucp1* knockout mice^35^. Accordingly, we observed a blunted HIF-1α protein accumulation after ISO stimulation of *Ucp1* knockdown brown adipocytes (Fig. 4). In addition, we observed that β-adrenergic stimulation stabilized HIF-1α in a manner dependent on mitochondrial ROS production (Fig. 4). Whether this ROS production is a result of mitochondrial uncoupling and/or hypoxia is not clear. However, since FCCP-induced mitochondrial uncoupling decreases ROS production in mitochondria isolated from brown adipocytes^38, 39^ it is unlikely that uncoupling by itself induces ROS production in response to β-adrenergic stimulation. Treatment with MitoQ is able to decrease hypoxia-induced HIF-1α stabilization but not proteasome inhibitor-induced HIF-1α stabilization^25^. This might indicate that the β-adrenergically induced ROS production is hypoxia-driven. Figure 8 summarizes our hypothesis on how β-adrenergic stimulation induces HIF-1α stabilization through mitochondrial uncoupling and ROS production.

We observed a reduced basal expression of *Glut1, Glut4*, *Ldha, Pfkl, Tpi1* and *Pgk1* after *Hif-1α* knockdown in both immortalized and primary brown adipocytes (Figs 5 and 6). The expression of these genes, except for *Glut4*, has been described to be under control of HIF-1α in embryonic stem cells and cancer cell lines^40–43^. The effect of *Hif-1α* knockdown on basal glycolytic gene expression is consistent with HIF-1α protein being present in brown adipocytes under these conditions (Figs 4–6). Furthermore, we observed a diminished expression of *Glut1*, *Ldha, Hk2, Pfkl, Tpi1* and *Pgk1* after β-adrenergic stimulation in *Hif-1α*-depleted adipocytes (Figs 5 and 6). Overall, HIF-1α seems to play an important role for both the basal and the β-adrenergically induced expression of glycolytic enzymes in brown adipocytes (Fig. 8).

A key ability of brown adipocytes is to produce heat through adaptive thermogenesis. If glucose is an important substrate for this process, glycolytic flux might be rate-limiting for the thermogenic potential. We observed decreased glucose uptake, lactate secretion, glycolysis and glycolytic capacity in *Hif-1α* knockdown cells (Fig. 7). Furthermore, knockdown of *Hif-1α* reduced the fold increase in ECAR and OCR after β-adrenergic stimulation in medium containing glucose as the only supplemented energy source (Fig. 7). Mitochondrial ROS scavenging also inhibited ECAR and OCR after β-adrenergic stimulation, indicating the importance of this upstream pathway (Fig. 7). This is in accordance with previous observations of decreased thermogenic potential *in vivo* in response to MitoQ treatment^24^.

The decreased glycolytic capacity after knockdown of HIF-1α in brown adipocytes is in agreement with other studies demonstrating glucose intolerance *in vivo* in response to decreased HIF-1α activity^20, 21^. On the other hand, several papers have shown increased insulin sensitivity in response to decreased HIF-1α activity^14–17^. The reason for this discrepancy in the literature is unclear. However, it should be noted that we are the first to study the role of HIF-1α on β-adrenergically stimulated glucose consumption. The mechanism by which HIF-1α regulates β-adrenergically and insulin stimulated glucose uptake differs, since hypoxia reduces phosphorylation and thereby activation of the insulin receptor^16^. The decrease in β-adrenergically stimulated ECAR and OCR after HIF-1α knockdown can likely be ascribed to the lower expression of glycolytic enzymes, as we did not observe a decreased expression of mitochondrial genes by knockdown of *Hif-1α* (Fig. 7) as have previously been reported^21^. Our findings show that HIF-1α positively impacts glucose metabolism in brown adipocytes. Accordingly, prolyl hydroxylase domain-containing protein 2 negatively affects glycolytic gene expression and glycolysis in white adipose tissue^20^. Mice with an adipocyte-specific knockout of *Hif-1α* are resistant to high fat diet-induced obesity, which correlated with increased ability of visceral WAT to oxidize palmitate^15^. Since HIF-1α can elicit opposite effects on adipocyte fatty acid and glucose oxidation, an intriguing possibility is that HIF-1α contributes to the substrate choice of activated brown adipocytes. More studies are required to decipher the exact function of HIF-1α in adipocyte metabolism in general and brown adipocyte metabolism in particular.

## Methods

### Materials

Collagenase type II (#C6885), Dulbecco’s Modified Eagle’s Medium (DMEM) (#D5030 and #D6429), dimethyl sulfoxide, glucose, 3-isobutyl-1-methylxanthine (IBMX), dexamethasone, isoproterenol (ISO), penicillin-streptomycin, pyruvate, MISSION siRNAs, sodium ascorbate and TRI Reagent were obtained from Sigma-Aldrich. Insulin was from Roche or kindly provided by Novo Nordisk A/S. Glucose-6-phosphate dehydrogenase, hexokinase, L-lactate dehydrogenase, ATP and NADP^+^ and NAD^+^ were from Roche. DMEM (#52100), fetal bovine serum (FBS), Lipofectamine RNAiMAX, 10% newborn calf serum, L-glutamine and Opti-MEM I Reduced Serum Medium were obtained from Life Technologies. Rosiglitazone was from Cayman Chemical. SensiFAST SYBR Lo-ROX Kit was from Bioline. HEPES was from Lonza, and carbonyl cyanide p-trifluoromethoxyphenylhydrazone (FCCP) was from Seahorse Bioscience. MitoQ was kindly provided by Dr. Mike Murphy^44^. Primary antibodies were against HIF-1α (#14179, Cell Signaling Technology), UCP1 (#Ab10983, Abcam) and transcription factor II B (TFIIB) (#sc-225, Santa Cruz Biotechnology). The secondary antibody was horse radish peroxidase-conjugated goat anti-rabbit (#P0448, Dako).

### Animals

Adipose tissues were obtained from 10-week-old male C57BL/6JBomTac mice (Taconic). Mice were single-caged at 22 °C and fed standard chow diet. For the cold/thermoneutral experiments, mice were housed at 4 °C for a period of 3 h up to 8 days, or at 30 °C (thermoneutrality) for 8 days (n = 6 in all groups, except in for the control group in which n = 12). Mouse experiments were approved by the Danish Animal Experimental Inspectorate. Care and handling of mice were in accordance with recommendations laid down by local authorities.

### Culture and differentiation of cell lines

The WT-1 cell line established by immortalization of brown pre-adipocytes from newborn mice with SV40 large T antigen was kindly provided by Dr. C. Ronald Kahn^23^. WT-1 cells were propagated in DMEM (Life Technologies) containing 10% FBS, 62.5 µg/ml penicillin and 100 µg/ml streptomycin. Two days after reaching confluence (designated day 0), differentiation was induced by addition of fresh propagation medium supplemented with 1 μM dexamethasone, 0.5 mM IBMX, 5 μg/ml insulin (Roche), and 0.5 μM rosiglitazone. At day 2, the medium was changed to medium with 5 μg/ml insulin and 0.5 μM rosiglitazone. From day 4, cells were cultured in propagation medium. The human brown pre-adipocyte cell model will be described elsewhere (Markussen *et al*., in review). Briefly, the cell model was generated by immortalization of the stromal vascular fraction of a deep neck adipose tissue biopsy with telomerase reverse transcriptase. Cell cultures were kept at 37 °C in a humidified atmosphere with 5% CO_2_. Cells were cultured in 6-well plates, except in siRNA experiments (see below). For the hypoxia experiments, cells were placed at 37 °C in a humidified atmosphere with 1% O_2_ and 5% CO_2_.

### Isolation and culture of primary adipocytes

Primary brown pre-adipocytes from interscapular, cervical and axillary BAT from 3–4 weeks old male NMRI mice (Taconic) were isolated and cultured as described^45^. The cell pellet was resuspended in culture medium and plated in 48-well or 6-well plates for ISO stimulation or siRNA transfections, respectively. For the ISO stimulation experiments, the medium was refreshed at days 1, 4, 6. For the siRNA transfection experiments, the medium was refreshed at days 1, 3, 4, 6 after isolation. Cultures were incubated in a humidified atmosphere of 8% CO_2_ at 37 °C.

### siRNA transfection

Mature adipocytes (on day 6 for WT-1 cells and day 8 for primary cultures) were reverse transfected with MISSION siRNA using Lipofectamine RNAiMAX diluted in Opti-MEM I Reduced Serum Medium as described^45^. The final concentrations of Lipofectamine and siRNA were 5 μl/ml and 50 nM, respectively, for WT-1 cells replated into 96-well and Seahorse plates, and 9 μl/ml and 90 nM, respectively, for primary adipocytes replated into 48-well plates. The siRNAs used targeting *Hif-1α* were SASI_Mm01_00070473 (siRNA 1) and SASI_Mm01_00070475 (siRNA 2) and the siRNA used for targeting *Ucp1* was SASI_Mm01_00067600^45^. The MISSION® siRNA Universal Negative Control #1 (SIC001) was used as control in all siRNA experiments.

### Seahorse measurements

Real-time measurements of oxygen consumption rate (OCR) and extracellular acidification rate (ECAR) were performed using the Seahorse XF96 Extracellular Flux Analyzer (Agilent Technologies). The cell culture medium was changed 1 h before the first measurement to DMEM (Sigma-Aldrich, #D5030) supplemented with 25 mM glucose with 10% fetal bovine serum (Fig. 3B), 2% BSA and 2 mM L-glutamine (Fig. 7C) or 5 mM glucose (Fig. 7D–K) and adjusted to pH 7.4. OCR and ECAR were measured under basal conditions and after injection of 0.1 µM ISO or 1 µM FCCP (Fig. 3), 10 mM glucose, 5 µM oligomycin and 50 mM 2-deoxyglucose (Fig. 7C) or 1 µM ISO (Fig. 7D–K). The Seahorse XF Glycolysis Stress Test was performed according to the manufacturer’s instructions (Agilent Technologies).

### Preparation of medium samples and quantitative measurements of glucose and lactate

Medium samples were prepared for quantitative measurements of glucose and lactate by mixing an equal amount of harvested medium from cultured adipocytes and cold 1.4 M perchloric acid (PCA). After vortexing and centrifuging (10 min, 20,000 g), the supernatant was transferred to clean tubes and pH adjusted to 7 by potassium hydroxide (KOH)/Hepes (2 M/0.1 M). The samples were then vortexed and centrifuged (10 min, 20,000 g) before the supernatant was transferred to clean tubes. The dilution factor of the medium was noted. Medium glucose and lactate were measured in cuvettes. Determination of medium glucose were performed by mixing 10 μl acid-precipitated medium, 10 µl glucose-6-phosphate dehydrogenase and 1000 µl Tris-MgCl_2_ buffer (0.2 M Tris, 2 mM MgCl_2_, 0.25 mg NADP^+^, 1.63 mg ATP, pH 7.5). Colorimetric measurements were then performed with an absorbance of 340 nm. After the initial measurement, 10 µl hexokinase was added and the mixture was incubated for 5–15 minutes before being measured again.

Measurements of medium lactate were performed by mixing 10 µl acid-precipitated medium with 1000 µl glycine-hydrazine buffer (38 mg glycine, 200 µl hydrazine 80%, 1.99 mg NAD^+^, pH 9). Colorimetric measurements were then performed with an absorbance of 340 nm. After the initial measurement, 10 µl L-lactate dehydrogenase was added and the mixture was incubated for 5–15 minutes before being measured again. For each sample, Equation 1 was used to calculate the glucose or lactate concentration:

1 $$\frac{{\rm{\Delta }}\mathrm{abs}\ast \mathrm{total}\,\mathrm{volume}\ast \mathrm{dilution}\,{\rm{factor}}}{6.22\ast \mathrm{light}\,\mathrm{path}\,({\rm{cm}})\ast \mathrm{sample}\,{\rm{volume}}}={\rm{concentration}}({\rm{mM}})$$

### Gene expression analysis

Total RNA was purified using TRI Reagent. Reverse transcription-quantitative PCR (RT-qPCR) was performed as previously described^46^, except that the SensiFAST SYBR Lo-ROX Kit was used. Primers used were: *CoxII* fwd-AATTGCTCTCCCCTCTCTACG, rev-GTAGCTTCAGTATCATTGGTGC; *Cs* fwd-CTCTTGGGAGCCAAGAACTC, rev-GCCTGCTCCTTAGGTATCAG; *Cyc1* fwd-CTACCCATGGTCTCATCGTG, rev-GGAAGAGCACACCTGCTTGT; *Glut1* fwd-GGACCCTGCACCTCATTG, rev-GCCACGATGCTCAGATAGG; *Glut4* fwd-ATCATCCGGAACCTGGAGG, rev-CGGTCAGGCGCTTTAGACTC; *Hif-1α* fwd-TCCATTTTCAACTCAGGACAC, rev-GGCAGTGATGGTAGGTTTCT; *Hk1* fwd-AGCATGGAGTCTGAGGTCTA, rev-CAGTACGGCCTCGTCTATTT; *Hk2* fwd-AGAGAACAAGGGCGAGGAG, rev-GGAAGCGGACATCACAATC; *Ldha* fwd-TAATGAAGGACTTGGCGGAT, rev-TTGGAGTTCGCAGTTACACA; *Pfkl* fwd-GCCTATCTCATCCAGCTACG, rev-CTTGCTACTCAGGATTCGGT; *Pfkp* fwd-AAGCTATCGGTGTCCTGACC, rev-TCCCACCCACTTGCAGAAT; *Pgc-1α* fwd-AGCCGTGACCACTGACAACGAG, rev-GCTGCATGGTTCTGAGTGCTAAG; *Pgk1* fwd-GAGCCTCACTGTCCAAACTA, rev-CTTTAGCGCCTCCCAAGATA; *Pkm1* fwd-CTGCTGTTTGAAGAGCTTGTG, rev-GAGTCACGGCAATGATAGGA; *Pkm2* fwd-TGCTGCAGTGGGGCCATTAT, rev-GAGTCACGGCAATGATAGGA; *Tbp* ^47^ fwd-ACCCTTCACCAATGACTCCTATG, rev-ATGATGACTGCAGCAAATCGC; *Tfam* fwd-CAAGTCAGCTGATGGGTATGG, rev-TTTCCCTGAGCCGAATCATCC; *Tpi1* fwd-TATGGAGGTTCTGTGACTGGA, rev-CGGTGGGAGCAGTTACTAAA; *Ucp1* fwd-AGCCGGCTTAATGACTGGAG, rev-TCTGTAGGCTGCCCAATGAAC.

### Whole cell extracts and immunoblotting

Cells stimulated with ISO were harvested at day 8 after initiation of differentiation (WT-1), eight days after isolation (primary brown adipocytes) or at day 12 of differentiation (human brown adipocytes), whereas cells transfected with *Hif-1α* siRNA were harvested four days after transfection. Preparation of whole-cell extracts and immunoblotting were done as described^47^.

### Statistical analyses

All data are presented as mean or mean of means + standard error of the mean (SEM). The cell culture data shown are the mean of three to five independent experiments. For gene expression studies in cell cultures, two to three wells were harvested at each time point and/or treatment in each experiment. All statistical tests of gene expression were performed on log-transformed data and as repeated measurements. Data in Figs 1, 3, 4A,F and H and 7H, were analyzed for statistical significance (P < 0.05) using one-way analysis of variance with post hoc testing of the means with Dunnett’s or Tukey’s correction for multiple comparisons. Data in Figs 2, 4B,C, 7C,E,G,I and K were analyzed for statistical significance (P < 0.05) using Student’s t-test. The results in Figs 5, 6 and 7A,B were analyzed by two-way analysis of variance with Tukey correction for multiple comparisons.

## Electronic supplementary material

Supplementary Figure S1
