# Supplementary material for: Regulation of glycolysis in brown adipocytes by HIF-1α
Source: Sci Rep. 2017 Jun 22;7:4052. doi: 10.1038/s41598-017-04246-y (PMC5481455; doi:10.1038/s41598-017-04246-y)

Regulation of glycolysis in brown adipocytes by HIF-1α

Astrid L. Basse, Marie S. Isidor, Sally Winther, Nina B. Skjoldborg, Maria Murholm, Elise S. Andersen, Steen B. Pedersen, Christian Wolfrum, Bjørn Quistorff & Jacob B. Hansen

Supplementary Information File

Supplementary Figure S1: Full-length blots for all immunoblots.

Figure 3E
HIF-1α

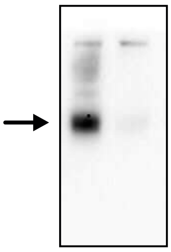

Figure 3E
TFIIB

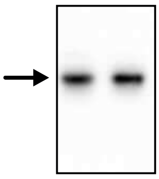

Figure 4C
HIF-1α

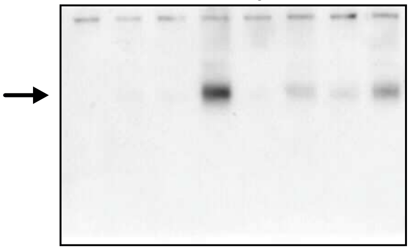

Figure 4C
TFIIB

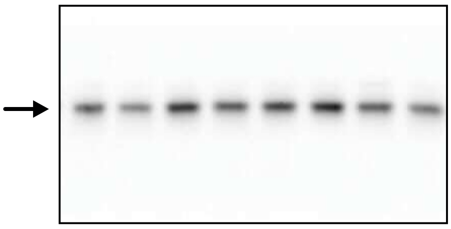

Figure 4D
HIF-1α

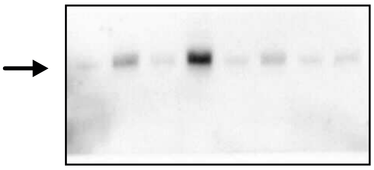

Figure 4D
TFIIB

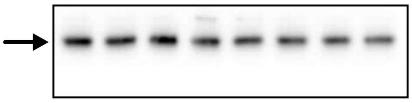

Figure 4E
HIF-1α

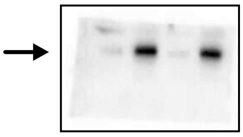

Figure 4E
TFIIB

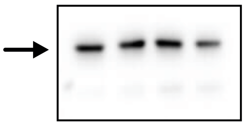

Figure 4G
HIF-1α

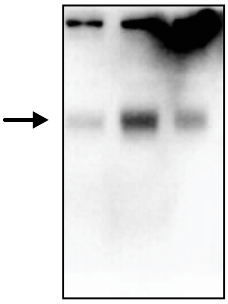

Figure 4G
TFIIB

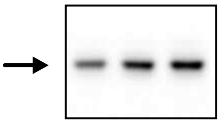

Figure 4I
HIF-1α

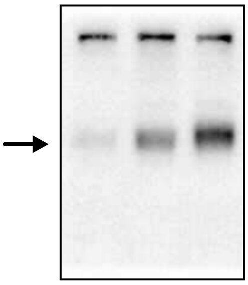

Figure 4I
TFIIB

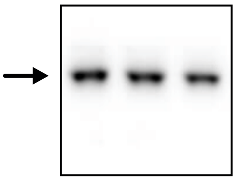

Figure 4J
HIF-1α

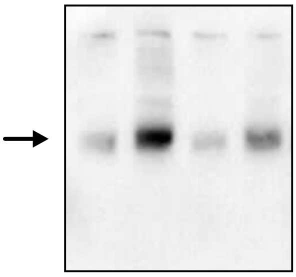

Figure 4J
UCP1

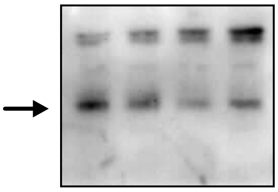

Figure 4J
TFIIB

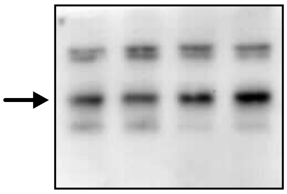

Figure 4K
HIF-1α

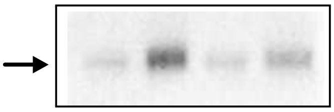

Figure 4K
UCP1

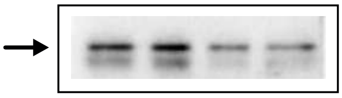

Figure 4K
TFIIB

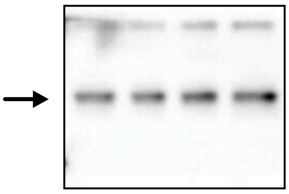

Figure 4L
HIF-1α

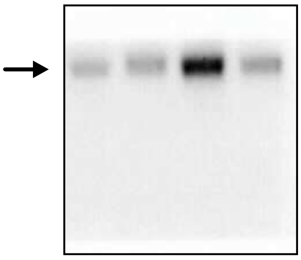

Figure 4L
TFIIB

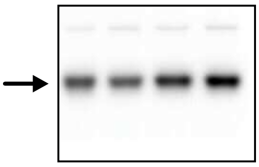

Figure 5B
HIF-1α

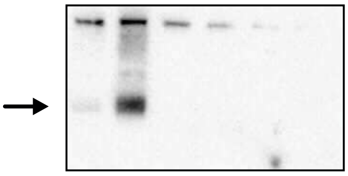

Figure 5B
TFIIB

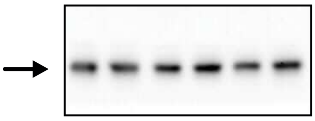

Figure 6B
HIF-1α

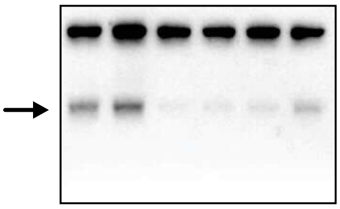

Figure 6B
TFIIB

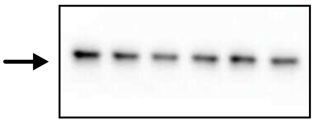

Supplement: Supplementary file 1 — Supplementary Figure S1 [file 41598_2017_4246_MOESM1_ESM.pdf]
